# Association between Dietary Vitamin E Intake and Esophageal Cancer Risk: An Updated Meta-Analysis

**DOI:** 10.3390/nu10070801

**Published:** 2018-06-21

**Authors:** Lingling Cui, Li Li, Yalan Tian, Fan Xu, Tianyi Qiao

**Affiliations:** Department of Nutrition and Food Hygiene, College of Public Health, Zhengzhou University, Zhengzhou 450001, Henan, China; lili01060209@163.com (L.L.); lhk0829@163.com (Y.T.); 18790670831@163.com (F.X.); 13253610181@163.com (T.Q.)

**Keywords:** dietary, vitamin E, tocopherol, esophageal cancer, meta-analysis

## Abstract

Epidemiological studies have provided ambiguous evidence on the association between vitamin E and esophageal cancer risk. To resolve this controversy, we performed this meta-analysis. The literature was searched by using Excerpta Medica Database (EMBASE), PubMed, the Web of Science, and the Cochrane Library from the inception to April 2018. A random effect model was utilized to calculate the odds ratio (OR) with the 95% confidence interval (95% CI). Twelve articles reporting 14 studies involving 3013 cases and 11,384 non-cases were included. By comparing the highest category with the lowest category of dietary vitamin E intake, we found that dietary vitamin E intake was inversely related to esophageal cancer risk (OR = 0.47, 95% CI: 0.36–0.60). Subgroup analysis revealed that dietary vitamin E intake had a significantly negative association with both the esophageal squamous cell carcinoma risk (OR = 0.29, 95% CI: 0.18–0.44) and the esophageal adenocarcinoma risk (OR = 0.66, 95% CI: 0.49–0.88). No study significantly affected the findings in the sensitivity analysis. Publication bias was discovered, however, the OR (95% CI) remained unchanged after the trim-and-fill analysis. This meta-analysis showed that the higher dietary vitamin E intake is associated with a lower esophageal cancer risk. However, the association still needs to be upheld by more large-scaled randomized controlled trials and prospective studies.

## 1. Introduction

Esophageal cancer is the eighth most common type of cancer worldwide. With an estimated 455,784 new cases and approximately 400, 169 deaths in 2012, esophageal cancer is the sixth most common cause of death from cancer. The highest mortality rates of esophageal cancer are in Eastern Asia (14.1 per 100,000) and Southern Africa (12.8 per 100,000) in men and in Eastern Asia (7.3 per 100,000) and Southern Africa (6.2 per 100,000) in women [1]. There are two main pathological subtypes of esophageal cancer: esophageal adenocarcinoma (EAC) and esophageal squamous cell carcinoma (ESCC). EAC is widespread in the western world, whereas ESCC is the predominant type for esophageal cancer worldwide [2,3]. EAC tends to occur in the distal esophagus from Barrett’s esophagus [3]. ESCC, however, has a tendency to occur in the squamous epithelium undergoing inflammatory changes [3]. Many possible factors cause esophageal cancer, including smoking, drinking, hot tea, red meat, poor oral health, low intake of fresh fruit and vegetables, low socioeconomic status, high body mass index (BMI), and *Helicobacter pylori* (*H. pylori*) infection [3,4]. Owing to the poor survival rate for esophageal cancer (overall ratio of mortality to incidence was 0.88) [1], nutritional prevention has been increasingly focused by scholars in recent decades.

Vitamin E is a fat-soluble vitamin including eight homologues: four tocopherols (α-tocopherol (T), β-T, γ-T, δ-T) and four tocotrienols (α-tocotrienol (TT), β-TT, γ-TT, δ-TT). A-tocopherol, the major active form of vitamin E with strong antioxidant properties, can eliminate reactive oxygen species, inhibit carcinogenesis and tumor growth, and stimulate cancer cell apoptosis [5,6,7,8]. Accordingly, it was presumed that higher dietary vitamin E intake was inversely associated with the risk of esophageal cancer. Up to now, many epidemiological studies have explored the association between vitamin E intake and the esophageal cancer risk. Some case–control studies reported that dietary vitamin E intake was inversely related with esophageal cancer risk [8,9,10,11,12], however, some studies were unable to find the inverse association [13,14]. To solve the divergence mentioned above, we performed this meta-analysis to systematically and quantitatively estimate the association between dietary vitamin E intake and esophageal cancer risk based on the relevant literature.

## 2. Materials and Methods

### 2.1. Search Strategy

Two investigators independently searched the electronic databases of Excerpta Medica Database (EMBASE), PubMed, the Web of Science, and the Cochrane Library up to April 2018. The following terms were used for the search: “(Vitamin E or tocopherol or tocopherols or α-Tocopherol or γ-Tocopherol or β-Tocopherol or δ-Tocopherol) and (esophageal or esophagus or oesophageal or oesophagus) and (neoplasm or neoplasms or cancer or cancers or carcinoma or tumor or tumors or tumour or tumours)”. In addition, the lists of references and reviews were also searched as potential supplements. The irrelevant articles were excluded by viewing the title, abstract, and full text. If one full-text could not be viewed or downloaded from the aforementioned electronic databases, we attempted to get the full-text by searching the online academic search and full-text delivery system or by contacting the corresponding author.

### 2.2. Study Selection

The inclusion criteria were the following terms: (1) cohort or randomized controlled trial (RCT) or case–control studies; (2) cases were diagnosed with esophageal cancer; (3) the dietary vitamin E intake was assessed by food frequency questionnaire and the vitamin E food composition database; (4) odds ratio (OR), hazard ratio (HR), or relative risk (RR) with their corresponding 95% confidence interval (CI) were reported or calculated; (5) detailed sample sizes were available; (6) articles were published in English; (7) as to dose–response analysis, the OR/HR/RR with 95% CI for esophageal cancer and person-years or the number of cases for each quantitative category of dietary vitamin E intake were available.

The following exclusion criteria were also considered: (1) nonhuman studies, review articles, letters to editor, or case reports; (2) cross-sectional studies; (3) studies where only vitamin E supplement use or serum vitamin E level were reported instead of dietary vitamin E intake; (4) studies where OR/RR/HR with 95% CI were not reported or calculated; (5) studies where detailed sample sizes were not reported.

### 2.3. Data Extraction and Quality Assessment

All data were extracted independently by two researchers and the ambiguities were resolved by a third investigator. The following information was extracted: (1) the last name of the first author; (2) publication year; (3) gender and age of participants; (4) the study period; (5) country; (6) source of control; (7) study design; (8) method of identifying cases; (9) the number of cases and non-cases; (10) assessment method of dietary vitamin E intake; (11) RR, OR, or HR (95% CI) for the maximizing adjusted model for the highest versus the lowest dietary vitamin E intake; (12) the adjusted covariance for multivariate analysis.

We assessed the methodological quality of included articles according to the instruction of Newcastle–Ottawa Scale (NOS) [15], including three quality parameters: selection, comparability, and outcome (for prospective studies) or exposure (for case–control studies). For the NOS score, one star means one point.

### 2.4. Statistical Analysis

OR, HR, and RR were used for analyzing dichotomous data in one meta-analysis. OR was a fair approximation of RR when the incidence of an event was rare. Since esophageal cancer incidence is small, OR and HR are theoretically similar to RR [16]. Therefore, OR was used only for pooling the dichotomous data in this meta-analysis. The heterogeneity among studies was estimated by Cochrane’s Q and *I*^2^ statistics. Generally, the 25%, 50%, and 75% of *I*^2^ values refer to low, moderate and high levels of heterogeneity, respectively [17]. If *I*^2^ > 50% and *p* < 0.05, a random-effect model was applied to pool OR (95% CI), if not, a fixed-effect model was used for pooling OR (95% CI) [18].

We performed meta-regression and subgroup analysis to explore the potential source of heterogeneity based on pathological type (ESCC, EAC, or mixed subtype), geographical distribution (America, Europe, or others), study type (case-control, or cohort study), source of control (population-based or hospital-based), sample size (≥500 or <500), the method for identifying esophageal cancer cases (pathological diagnoses or cancer registries), dietary vitamin E assessment method (validated food frequency questionnaire (FFQ) or unvalidated FFQ), quality score (>5 or ≤5), OR/HR/RR adjusted for age (yes or no), gender (yes or no), smoking (yes or no), drinking (yes or no), and BMI (yes or no), respectively. In addition, sensitivity analyses were also performed to explore the potential study, which greatly affected the robustness of the result. Due to different definitions of exposure categories in separate studies, a fixed-effect dose–response analysis was performed to quantitatively estimate the potential association between the increment per day for dietary vitamin E intake and the esophageal cancer risk. We recalculated the pooled OR of esophageal cancer for every 3 mg/day increment of dietary vitamin E intake through previously described methods [19,20]. Furthermore, a non-linear dose–response analysis was likewise performed by using restricted cubic splines with three knots at fixed percentiles (10%, 30% and 50%) of the distribution of exposure [21,22]. We calculated the overall *p*-value by testing that the two regression coefficients were simultaneously equal to zero. We also calculated *p*-values for non-linearity by testing that the coefficient of the second spline was equivalent to zero. The details of this method have been cited previously [23,24]. The aforementioned method involves the following items: (1) the number of cases and person-years or non-cases and the RRs or ORs with the variance estimates for at least three quantitative exposure categories; and (2) the median or mean level of these exposures in each category (if ranges was reported, mean levels were calculated by averaging the lower and upper boundary; if the lower boundary of the lowest category was not reported, we set zero as the lower boundary; if the upper boundary of the highest category was not reported, we added the same amplitude with the closest category to the lower boundary as the upper boundary) [25,26].

Finally, Egger’s and Begg’s test were performed to assess the potential publication bias. If both of *p* values for Egger’s test and Begg’s test were less than 0.05 (*p* < 0.05), it indicated the publication bias existed [27]. If the publication bias existed, we used the “trim and fill” method to add the potentially missing studies for adjustment for the funnel plot’s asymmetry [28,29], and observed the variation of pooled OR (95% CI). If the pooled OR (95% CI) changed weakly or remained unchanged, it indicated that the result was robust. All the statistical analyses were performed by using Stata 11.2 software (Stata Corp Lakeway Drive, College Station, TX, USA), and a two-sided *p* value < 0.05 was considered statistically significant.

## 3. Results

### 3.1. Literature Search, Study Characteristics, and Quality Assessment

**Figure 1:**
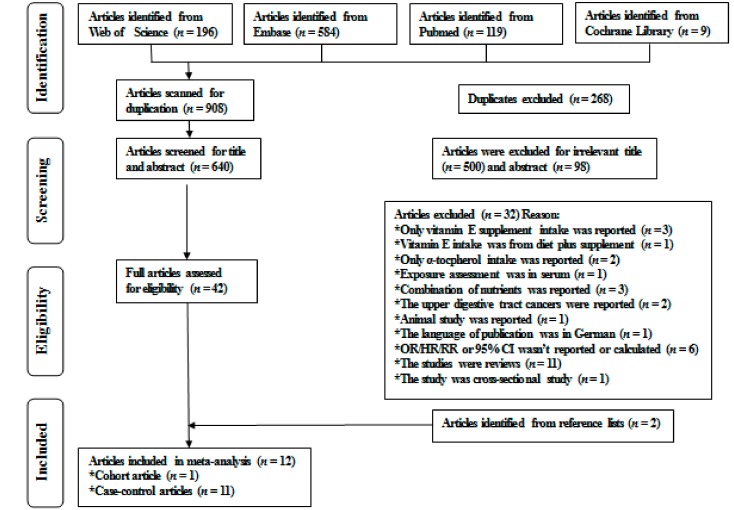
Flow chart of the electronic search for eligible articles. OR: odds ratio; HR: hazard ratio; RR: relative risk; CI: confidence interval.

A total of 599 articles, 256 articles, 123 articles, and 29 articles were obtained from EMBASE, PubMed, the Web of Science, and the Cochrane Library, respectively. After removing 278 duplicate studies, 729 articles remained for viewing the titles and abstracts, and then 42 articles remained for review of the full text for eligibility. Among them, three articles reported vitamin E supplementation but did not report dietary vitamin E intake in relation to esophageal cancer, one article reported vitamin E intake from diet plus supplements, two articles reported the correlation between dietary α-tocopherol intake and esophageal cancer, one article reported serum vitamin E level in relation to esophageal cancer, three articles reported combination nutrients in relation to esophageal cancer, two articles reported vitamin E intake and upper digestive tract cancer, one article was an animal study, one article was published in German, six articles did not report OR/HR/RR or 95% CI, 11 studies were reviews, and one article was a cross-sectional study. Besides, two additional articles were obtained by screening the lists of references [9,30]. Finally, 12 articles reported 14 studies involving 3013 cases and 11,384 non-cases were selected for the meta-analysis [8,9,10,11,12,13,14,30,31,32,33,34]. The detailed processes of screening articles are presented in Figure 1.

Among these articles, one article was a cohort study [34] and 11 articles were case-control studies [8,9,10,11,12,13,14,30,31,32,33] in the meta-analysis. These studies were conducted in Europe [9,10,14,31,33,34], America [13,30,32], Asia [11,12], and Oceania [8]. Dietary vitamin E intakes were assessed by a validated food frequency questionnaire (FFQ) [8,9,10,11,12,13,14,30], which was more applicable than the unvalidated FFQ [31,32,33,34]. The identification of esophageal cancer cases was based on cancer registries [9,13,30] and the pathological diagnoses [8,10,11,12,14,31,32,33,34]. The detailed characteristics of included studies are presented in Table 1. For the quality assessment of the included studies, four articles involving five studies were granted for points ≤5 [31,32,33,34], eight articles involving nine studies were given points >10 [8,9,10,11,12,13,14,30], and details of the quality assessment are presented in Supplementary Tables S1 and S2.

### 3.2. Meta-Analysis of Dietary Vitamin E Intake and the Esophageal Cancer Risk

**Figure 2:**
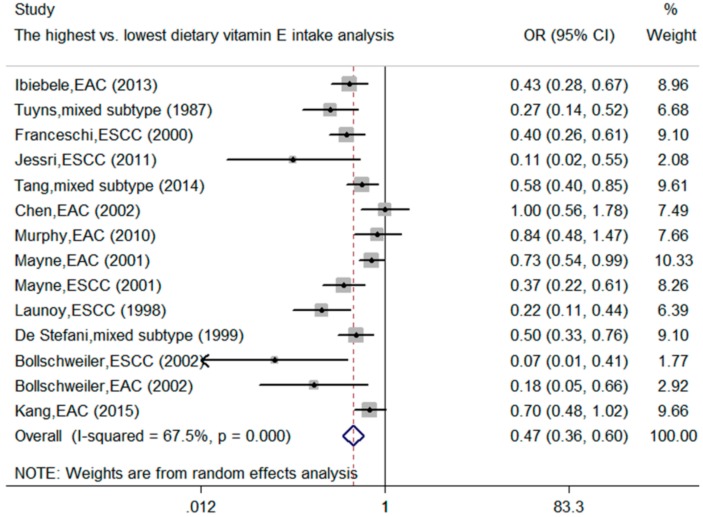
The forest plot for the meta-analysis on dietary vitamin E intake and the esophageal cancer risk. ESCC: esophageal squamous cell carcinoma; EAC: esophageal adenocarcinoma; mixed subtype: did not report the specific subtype of esophageal cancer.

Twelve articles involving 14 studies on dietary vitamin E intake and the esophageal cancer risk were included in this meta-analysis. The pooled OR of the esophageal cancer risk for the highest vs. lowest intake of dietary vitamin E was calculated. In a comprehensive analysis, we found that dietary vitamin E intake was inversely associated with the esophageal cancer risk (OR = 0.47, 95% CI: 0.36–0.60) with significant heterogeneity (*I*^2^ = 67.5%; *p* < 0.001) (Figure 2, Table 2).

### 3.3. Meta-Regression and Subgroup Analysis

Since significant heterogeneity was found in this meta-analysis; meta-regression and subgroup analysis were performed to explore the source of the between-study heterogeneity. Meta-regression analysis found adjustment for variables like age (*p* = 0.044), gender (*p* = 0.017), smoking (*p* = 0.044), and BMI (*p* = 0.017) was the possible source of the heterogeneity. The following covariance such as geographical distribution (*p* = 0.475), study type (*p* = 0.403), source of control (*p* = 0.433), sample size (*p* = 0.821), pathological type (*p* = 0.072), the method of identifying esophageal cancer cases (*p* = 0.463), dietary vitamin E assessment method (*p* = 0.394), quality score (*p* = 0.355), and adjustment for drinking (*p* = 0.261) did not explain the between-study heterogeneity.

In the subgroup analysis, when stratified by adjustment for variables like age, gender, smoking, and BMI, no statistical heterogeneity was found in the studies without adjustment for variables like age (*I*^2^ = 0.0%, *p* = 0.398), gender (*I*^2^ *=* 0.0%, *p* = 0.556), smoking (*I*^2^ = 0.0%, *p* = 0.398), and BMI (*I*^2^ = 0.0%, *p* = 0.556). When controlled by pathological type, heterogeneity decreased in the EAC cases (*I*^2^ = 53.3%, *p* = 0.057), ESCC cases (*I*^2^ = 43.3%, *p* = 0.133), and mixed subtype cases (*I*^2^ = 48.7%, *p* = 0.142). In general, in the cases with the highest vs. the lowest category of the dietary vitamin E intake, dietary vitamin E has a rather inverse association with both the risk of EAC (OR = 0.66, 95% CI: 0.49–0.88) and the risk of ESCC (OR = 0.29, 95% CI: 0.18–0.44). The detailed results on subgroup analysis are shown in Table 2.

### 3.4. Sensitivity Analysis, Publication Bias Analysis, and Trim and Fill Analysis

**Figure 3:**
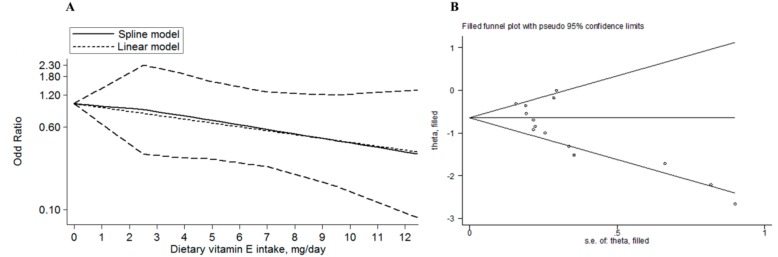
(**A**)The dose-response analyses for the association between dietary vitamin E intake and the esophageal cancer risk. The solid line and the long dash line represent the pooled OR and corresponding 95% CI. The short dash line represents the linear association; (**B**) The funnel plot with trim and fill analysis for studies on the association between dietary vitamin E intake and the esophageal cancer risk.

A total of four articles reporting five studies involving 970 cases and 2225 non-cases [2,8,14,33] were included in the dose–response analyses based on the relevant criteria mentioned in the method of statistical analysis. Among the four articles [2,8,14,33], the dietary vitamin E intake per day was defined mg/day, except for Murphy’s article [14], which was defined intake in µg/day. Therefore, we turned the µg/day to mg/day by dividing 1000. Overall analysis, no heterogeneity was found (*p* = 0.987), no significant non-linear dose–response relationship was found (pnon-linearity = 0.864), but a marginally linear dose–response relationship was found for 3 mg/day increment of dietary vitamin E intake and the risk of esophageal cancer (OR = 0.78, 95% CI: 0.57–1.06, plinearity = 0.114) (Figure 3A). For the sensitivity analysis, no one specific study significantly affected the pooled OR (95% CI) (Supplementary Figure S1). Both Egger’s test and Begg’s test found publication bias existed (*p* = 0.008, and *p* = 0.005, respectively). Then, trim and fill analysis was performed for the adjustment of funnel plot asymmetry; no missing studies were added and the adjusted OR was 0.47 (95% CI: 0.36–0.60), which showed the pooled OR (95% CI) remained unchanged. The funnel plot of trim and fill analysis was shown in Figure 3B.

## 4. Discussion

Up to now, epidemiology studies have provided ambiguous evidences for the relationship between dietary vitamin E intake and the esophageal cancer risk. To solve the divergence, we performed a meta-analysis on the association between dietary vitamin E intake and esophageal cancer risk (including EAC and ESCC). We found that the higher dietary vitamin E intake was related to a lower esophageal cancer risk, especially for ESCC. The aforementioned findings were in line with many other studies of dietary vitamin E intake and various cancer risks. One published dose–response meta-analysis has suggested that bladder cancer risk decreased by 17% for every 10 mg/day increase of dietary vitamin E intake (RR = 0.83, 95% CI: 0.72–0.95) [35]. Another meta-analysis focused on the association between dietary antioxidant vitamins intake and gastric cancer, which showed the higher dietary vitamin E intake decreased the risk of gastric cancer by 35% (OR = 0.65, 95% CI: 0.57–0.74), and the gastric cancer risk decreased by 21% for every 15 mg/day increase of dietary vitamin E intake (OR = 0.79, 95% CI: 0.66–0.94) [36]. The findings of one latest meta-analysis containing 11 studies (10 prospective studies and one case–control study) also showed that the dietary vitamin E intake was inversely associated with the risk of lung cancer (RR = 0.86, 95% CI: 0.74–0.99) [37]. The findings of one recent meta-analysis containing 11 studies (10 prospective studies and one case–control study) also showed that the dietary vitamin E intake was inversely associated with the risk of lung cancer (RR = 0.86, 95% CI: 0.74–0.99) [37]. Therefore, the combination of the meta-analyses mentioned above and our study further suggested that dietary vitamin E intake was inversely associated with many cancer risks, which might provide relevant population-based evidence for vitamin E preventing tumorigenesis, including in esophageal cancer.

In 2007, Kubo’s meta-analysis explored the association between antioxidant intake (including vitamin E) and the risk of esophageal and gastric cardia adenocarcinoma, and showed a marginally inverse association between vitamin E intake and EAC risk (OR = 0.80, 95% CI: 0.63–1.03), but did not report the relationship between vitamin E intake and ESCC risk [38]. In Kubo’s research, a total of three case–control studies on the association between vitamin E intake and the EAC risk were included, two of which were on dietary vitamin E intake and the EAC risk and one of which was on dietary α-tocopherol and EAC risk. α-tocopherol is one of vitamin E’s homologues, but we mainly take in total vitamin E not just α-tocopherol from various foods such as cereal grains, nuts, particularly vegetable oils [39]. The dietary vitamin E intake was calculated by using the following formulas that came from the literature of Yang Yue et al. [40]: “α-tocopherol equivalent (α-TE, mg) = 1.0 × α-tocopherol (mg) + 0.5 × β-tocopherol (mg) + 0.1 × γ-tocopherol (mg) + 0.02 × δ-tocopherol (mg) + 0.3 × α-tocotrienols”, or “ vitamin E (mg) = α-tocopherol (mg) + (β + γ)-tocopherol (mg) + δ-tocopherol (mg)” [41]. Importantly, dietary α-tocopherol intake is not equivalent to dietary vitamin E intake. Therefore, Carman’s and Terry’s studies were excluded in our meta-analysis because of only reporting dietary α-tocopherol intake [40,42].

The present meta-analysis showed that higher vitamin E intake is negatively associated with the risk of esophageal cancer. This may be explained by the strong oxidative properties of vitamin E [43,44]. One previous study showed that vitamin E could inhibit DNA oxidative damage and lipid peroxidation by reducing the level of endogenous reactive oxygen species, and thereby prevented gene mutations as well as tumor formation [45]. Additionally, in N-nitrosomethylbenzylamine (NMBZA)-treated rats, 8OH-dG was significantly increased in various esophageal lesions, whereas early continuous supplementation of vitamin E reduced 8OH-dG in each category of lesions [46]. In vivo, vitamin E may also suppress NMBZA-induced carcinogenesis in rat esophagus by blocking activation of nuclear factor-kappa B (NFκB) and abnormal metabolic pathway of arachidonic acid [47].

Based on the results of meta-regression and subgroup analysis, we found that the possible sources of between-study heterogeneity for this meta-analysis were adjustment for variables like age, gender, smoking, and BMI. As we know, the incidence of esophageal cancer increases with age, and the incidence of esophageal cancer worldwide in males is more than double that in females (2.4:1). Furthermore, one meta-analysis of 12 studies showed that about 68% esophageal cancer cases were attributed to smoking (RR = 3.01, 95% CI: 2.30–3.94) [48]. As for BMI, it was widely used for determining obesity and overweight. When the dose increased by per 5 kg/m^2^ for BMI, the esophageal cancer risk was enhanced by approximately 10% (RR = 1.11, 95% CI: 1.09–1.14) [49,50]. The aforementioned findings may respectively explain the reasons for adjustment for variables like age, gender, smoking, and BMI as the potential sources of heterogeneity for this meta-analysis. Besides, when stratified by pathological type, we found the heterogeneity was reduced, which revealed that pathological type was also the potential source of heterogeneity. The higher dietary vitamin E intake has a negative correlation with the risk of ESCC, which were more than the negative correlation double those for the EAC risk.

To our knowledge, the present meta-analysis had several advantages Firstly, the study was a quantitative and comprehensive dose–response analysis to evaluate the association between dietary vitamin E intake and the risk of esophageal cancer. Secondly, meta-regression and subgroup analysis were simultaneously performed to explore the potential sources of heterogeneity. Thirdly, sensitive analysis found no one specific study significantly affected the pooled OR (95% CI). Fourthly, trim and fill analysis did not find potentially missing articles, and the pooled OR (95% CI) was maintained after the adjustment of the asymmetry of the funnel plot, which revealed that our findings might be robust and sound.

However, several limitations should also be taken into consideration. Firstly, most of the eligible studies were case–control studies, which were prone to cause recall bias and selection bias. Secondly, dietary vitamin E intakes were assessed by validated FFQs [8,9,10,11,12,13,14,30], and dietary vitamin E intakes were assessed by unvalidated FFQs [31,32,33,34]. The former was more applicable than the latter, and 33% articles used not-validated FFQ, which might affect the estimation of the dietary vitamin E intake. Thirdly, only a few studies were included in the dose–response analysis, and the corresponding sample size was small. Therefore, the association between the increase of per day dietary vitamin E intake and the esophageal cancer risk should be explored by using more usable data in the future investigation. Last but not least, there was a higher mortality rate for esophageal cancer in Eastern Asia according to the data from GLOBOCAN 2012, but most studies were conducted in Europe and America, and only few studies were performed in Asia. This may induce regional restrictions to the findings, and they may not be generalized to Asian population very well. Therefore, studies on dietary vitamin E and esophageal cancer research should be expanded, especially in areas with high incidence of esophageal cancer, such as in the Asian region. The association between dietary vitamin E intake and esophageal cancer risk should be researched in more areas worldwide.

## 5. Conclusions

In summary, our study suggests that dietary vitamin E intake is inversely associated with the esophageal cancer risk. Considering the limitations of the included studies, more large-scale prospective studies and randomized controlled trials are still needed to confirm our results, though our findings could provide certain evidence for esophageal cancer prevention.

## Figures and Tables

**Table 1:** Characteristics of included studies on the association between dietary vitamin E intake and esophageal cancer.

The First Author, Publication Year	Gender, Age, Study Period	Country, Source of Control, Study Design	Method of Identifying Case, the Number of Case/Non-Case	Methods of Dietary Vitamin E Intake Assessment	OR/HR/RR (95% CI) for Highest vs. Lowest Dietary Vitamin E Intake	Adjustment for Confounders
Ibiebele, 2013 [8]	Both sexes, (18–79) years, 2002–2005	Australian, population-based case-control	Pathological diagnoses, 299/1507	Validated-FFQ-135 items	0.43 (0.28, 0.67) for EAC	Age, gender; education, BMI, frequency of heartburn or acid reflux symptoms, smoking, alcohol, NSAID use, total energy intake
Tuyns, 1987 [9]	Both sexes, NA, NA	France, population-based case-control	Cancer registries, 743/1975	Validated-FFQ-40 items	0.27 (0.12, 0.45) for mixed subtype ^a^	Age, alcohol, and smoking
Franceschi, 2000 [10]	Both sexes, (35–77) years, 1992–1997	Italian, hospital-based case-control	Pathological diagnoses, 304/743	Validated-FFQ-78 items	0.40 (0.30, 0.70) for ESCC	Age, gender, area of residence, education, physical activity, BMI, smoking, alcohol drinking and non-alcohol energy
Jessri, 2011 [11]	Both sexes, (40–75) years, NA	Iran, hospital-based case-control	Pathological diagnoses, 47/96	Validated-FFQ-125 items	0.11 (0.03, 0.74) for ESCC	Age, sex, gastroesophageal reflux disease symptoms, BMI, smoking, physical activity, education
Tang, 2014 [12]	Both sexes, (48.8–72.4) years, 2008–2009	China, hospital-based case-control	Pathological diagnoses, 350/380	Validated-FFQ-137 items	0.58 (0.40, 0.85) for mixed subtype ^a^	Age, gender, education, BMI, energy intake, smoking, alcohol, family history of cancer
Chen, 2002 [13]	Both sexes, (42.2–74.7) years, 1992–1994	America, population-based case-control	Cancer registries, 124/449	Validated-FFQ-60 items	1.00 (0.60, 1.90) for EAC	Age, age squared, gender, respondent type, BMI, alcohol, tobacco, education, family history of cancers, vitamin supplement
Murphy, 2010 [14]	Both sexes, (53–75) years, 2002–2004	Ireland, population-based case-control	Pathological diagnoses, 224/256	Validated-FFQ-188 items	0.84 (0.48, 1.47) for EAC	Age, sex, BMI, energy intake, smoking, education, occupation, alcohol, NSAID use, gastroesophageal reflux disease, location, *H. pylori* infection
Mayne, 2001 [30]	Both sexes, (30–79) years, 1996–1999	America, population-based case-control	Cancer registries, 282/687 for EAC; 206/687 for ESCC	Validated-FFQ-104 items	0.73 (0.54, 1.00) for EAC; 0.37 (0.22, 0.60) for ESCC	Sex, site, age, race, proxy status, income, education, BMI, cigarettes, alcohol, and energy intake
Launoy, 1998 [31]	Male, NA, 1991–1994	France, hospital-based case-control	Pathological diagnoses, 208/399	Non-validated-FFQ-39 items	0.22 (0.11, 0.44) for ESCC	Interviewer, age, smoking, beer, aniseed aperitives, hot Calvados, whisky, total alcohol, total energy intake and other significant food groups, PUFA, SFA
De Stefani, 1999 [32]	Both sexes, (30–89) years, 1996–1997	Uruguay, hospital-based case-control	Pathological diagnoses, 66/393	Not-validated-FFQ-64 items	0.50 (0.30, 0.70) for mixed subtype ^a^	Age, sex, residence, urban/rural status, education, BMI, smoking, alcohol and energy intake
Bollschweiler, 2002 [33] ^b^	Male, (56–62.6) years, 1997–2000	Germany, population-based case-control	Pathological diagnoses, 52/50 for ESCC; 47/50 for EAC	Not-validated-FFQ-1100 items	0.07 (0.01, 0.34) for ESCC; 0.18 (0.05, 0.67) for EAC	None
Kang, 2015 [34]	Both sexes, (40–65) years, NA-2008 ^c^	UK, cohort	Pathological diagnoses, 61/3712	Not-validated-FFQ	0.70 (0.48, 1.01) for EAC	Age, gender, BMI and smoking

EAC: esophagus adenocarcinoma; FFQ: food frequency questionnaire; OR: odds ratio; BMI: body mass index; NSAID: nonsteroidal anti-inflammatory drug; NA: not applied; ESCC: esophageal squamous cell carcinoma; RR: relative risk; *H.pylori*: *Helicobacter pylori*; PUFA: polyunsaturated fatty acid; SFA: saturated fatty acids; HR: hazard ratio; ^a^ mixed subtype: did not report specific subtype; ^b^ this study did not report the ORs (95% CI) of ESCC and EAC for the highest vs. lowest category of dietary vitamin E intake, so we recalculated the ORs (95% CI) based on usable data; ^c^ did not report the number of the items.

**Table 2:** Overall and subgroup analysis of the association between dietary vitamin E intake and the risk of esophageal cancer.

Outcome of Interest	No. of Studies	No. of Cases/Non-Cases	ORs (95% CIs)	*p* for Test	Heterogeneity Test	
					*I*^2^ (%)	*p_het_*
Dietary Vitamin E	14	3013/11,384	0.47 (0.36, 0.6)	<0.001	67.5	<0.001
Pathological type						
EAC	6	1037/6661	0.66 (0.49, 0.88)	0.005	53.5	0.057
ESCC	5	817/1975	0.29 (0.18, 0.44)	<0.001	43.4	0.133
Mixed subtype *	3	1159/2748	0.46 (0.32, 0.68)	<0.001	48.7	0.142
Geographic location						
Europe	7	1639/7185	0.37 (0.23, 0.60)	<0.001	73.9	0.001
America	4	678/2216	0.60 (0.41, 0.88)	0.008	65.8	0.032
Others ^†^	3	696/1983	0.44 (0.27, 0.73)	0.001	55	0.108
Study design						
Case-control	13	2952/7672	0.44 (0.37, 0.58)	<0.001	68	<0.001
Cohort	1	61/3712	0.7 (0.48, 1.02)	0.06	NA	NA
Source of control						
Population	9	2038/9373	0.51 (0.36, 0.71)	<0.001	70.3	0.001
Hospital	5	975/2011	0.40 (0.28, 0.58)	<0.001	57.1	0.054
Sample size						
<500	6	497/4557	0.43 (0.25, 0.72)	0.002	68.5	0.007
≥500	8	2516/6827	0.47 (0.35, 0.63)	<0.001	70.3	0.001
Method of case identified						
Pathological diagnoses	10	1658/7586	0.44 (0.32, 0.59)	<0.001	63.3	0.004
Cancer registries	4	1355/3798	0.53 (0.32, 0.9)	0.02	78.2	0.003
Dietary vitamin E assessment method						
Validated-FFQ	9	2579/6780	0.52 (0.38, 0.68)	<0.001	66.3	0.003
Unvalidated-FFQ	5	434/4604	0.34 (0.19, 0.62)	<0.001	74.4	0.004
Quality score						
>5	9	2579/6780	0.51 (0.38, 0.68)	<0.001	66.3	0.003
≤5	5	434/4604	0.34 (0.19, 0.62)	<0.001	74.4	0.004
Adjusted factors						
Age						
Yes	12	2914/11,284	0.50 (0.39, 0.64)	<0.001	65.9	<0.001
No	2	99/100	0.13 (0.05, 0.37)	<0.001	0	0.398
Gender						
Yes	10	1963/8910	0.56 (0.45, 0.70)	<0.001	56.5	0.014
No	4	1050/2474	0.22 (0.14, 0.34)	<0.001	0	0.556
Smoking						
Yes	12	2914/11,284	0.50 (0.39, 0.64)	<0.001	65.9	0.001
No	2	99/100	0.13 (0.05, 0.37)	<0.001	0	0.398
Drinking						
Yes	10	2806/7476	0.50 (0.39, 0.64)	<0.001	65.9	0.002
No	4	207/3908	0.21 (0.06, 0.73)	0.014	77.9	0.004
BMI						
Yes	10	1963/8910	0.56 (0.45, 0.70)	<0.001	56.5	0.014
No	4	1050/2474	0.22 (0.14, 0.34)	<0.001	0	0.556

No.: number; ORs: odds ratios; *p_het_*: *p*-value for heterogeneity within each subgroup; EAC: esophagus adenocarcinoma; ESCC: esophageal squamous cell carcinoma; FFQ: food frequency questionnaire; BMI: body mass index; * Mixed cancer: did not reported specific subtype; ^†^ Asia, Oceania.

## Supplementary Materials

The following are available online at http://www.mdpi.com/2072-6643/10/7/801/s1. Figure S1: The sensitive analyses for studies on the association between dietary vitamin E intake and the esophageal cancer risk. Table S1: Methodological quality of case-control studies included in the meta-analysis, Table S2: Methodological quality of prospective studies included in the meta-analysis.
