# Supplementary material for: Association between Dietary Vitamin E Intake and Esophageal Cancer Risk: An Updated Meta-Analysis
Source: Nutrients. 2018 Jun 21;10(7):801. doi: 10.3390/nu10070801 (PMC6073499; doi:10.3390/nu10070801)
Supplement: Supplementary file 1 [file nutrients-10-00801-s001.pdf]

Supplementary Table 1. Methodological quality of case-control studies included in the meta-analysis*.

First author, publication year	Cases identified for cancer registers	Representativeness of cases	Control subjects were from population§	Definition of control subjects	Control for important factor or additional factor†	Exposure assessment&	Same method of ascertainment for all subjects	No heterogeneity in the response rate for case and control groups ‡
Ibiebele, 2013 (9)	★	★	★	★	★	★★	★	-
Tuyns, 1987 (10)	★	★	★	★	-	★★	★	-
Franceschi, 2000 (11)	★	★	-	★	-	★★	★	-
Jessri, 2011 (12)	★	★	-	★	★	★★	★	-
Tang, 2014 (13)	★	★	-	★	★	★★	★	-
Chen, 2002 (14)	★	★	★	★	★	★★	★	-
Murphy, 2010 (15)	★	★	★	★	★★	★★	★	-
Mayne, 2001 (31)	★	★	★	★	-	★★	★	-
Launoy, 1998 (32)	-	★	-	★	★	-	★	-
De Stefani, 1999 (33)	★	★	-	★	-	★	★	-
Bollschweiler, 2002 (34)	-	★	★	★	-	-	★	★

*A study could be granted for a maximum of one point for each category except for the category of control for important factor or additional factor, and exposure assessment. § Population-based case-control studies were granted for one point. † The column received a maximum of 2 points. Two points were granted for studies controlled for both gastroesophageal reflux disease and other important variables such as H. p. infection, family history of cancers, and vitamin supplement. & Maximum of 2 points was granted for this column. Two points were granted for studies that esophageal cancer case was identified for caner registries and dietary vitamin E intake was assessed by validated Food Frequency Questionnaire (FFQ). ‡ One point was assigned for studies that no significant difference was found in the response rate between control groups and case groups

Supplementary Table 2. Methodological quality of prospective studies included in the meta-analysis*.

First author, publication year	Representativeness of the exposed cohort	Unexposed cohort was from population	Ascertainment of exposure	Outcome of esophageal cancer not present at start of study	Control for important factor or additional factor†	Assessment of esophageal cancer&‡	During of follow-up ≥10 year ‡	Follow-up rate ≥ 75%‡
Kang, 2015 (35)	★	★	★	★	-	-	-	-

* A study could be assigned a maximum of one point for each category except for the category of control for important factor or additional factor and assessment of outcome. † The column was granted for a maximum of 2 points. Two points were assigned for studies controlled for both gastroesophageal reflux disease and other important variables such as H. p. infection, family history of cancers, and vitamin supplement. & Maximum of 2 points was granted for this column. Two points were granted for studies that investigators were blind for group and esophageal cancer cases were identified for caner registries. ‡A point was granted for the study that during of follow-up was more than 10 year. §A cohort study with a follow-up rate > 75% could receive one point.

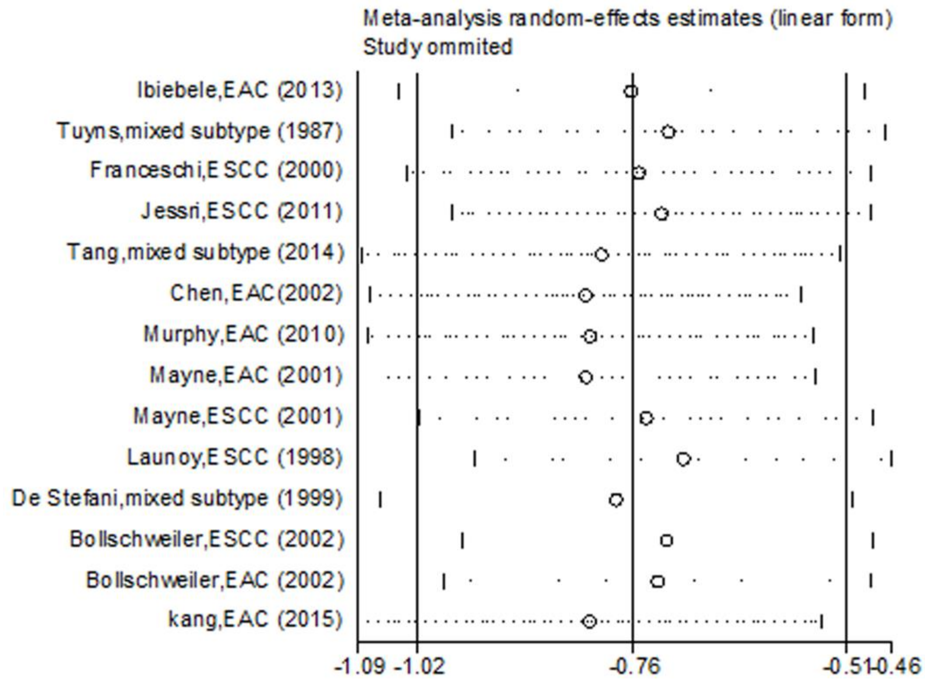

Supplementary Figure 1. The sensitive analyses for studies on the association between dietary vitamin E intake and the esophageal cancer risk. ESCC: esophageal squamous cell carcinoma; EAC: esophageal adenocarcinoma; mixed subtype: didn't report the specific subtype of esophageal cancer.
